# Autism in Africa: prevalence, diagnosis, treatment and the impact of social and cultural factors on families and caregivers: a review

**DOI:** 10.1097/MS9.0000000000001107

**Published:** 2023-07-24

**Authors:** Nicholas Aderinto, Deji Olatunji, Oluwatobi Idowu

**Affiliations:** a Department of Medicine and Surgery, Ladoke Akintola University of Technology, Ogbomoso, Cameroon; b Department of Medicine and Surgery, University of Ilorin, Ilorin, Nigeria; c Department of Psychiatry, Chesire and Wirral Partnership NHS Foundation Trust, Chester, UK

**Keywords:** Africa, autism, neurology

## Abstract

This paper presents a narrative review of current knowledge on autism in Africa, including prevalence, diagnosis, treatment and the impact of social and cultural factors on families and caregivers. The prevalence of autism in Africa is estimated to be similar to that in other regions. However, diagnosis and treatment access remain limited due to various challenges, such as a shortage of specialised healthcare professionals and resources, a lack of awareness and understanding of autism spectrum disorder (ASD) among healthcare providers, and cultural stigma surrounding mental health and developmental disorders. Alternative therapies are commonly used with other therapies, but their effectiveness is often unproven. The impact of ASD on families and caregivers in Africa is significant, with many facing challenges in accessing support services and coping with stigma. Efforts are being made to increase awareness and reduce the stigma around ASD in African communities, but more research is needed on effective interventions and culturally appropriate treatments. Policy recommendations include increasing resources and training for healthcare professionals, improving access to evidence-based interventions and promoting community awareness and support. With improved understanding and investment, the quality of life of individuals with ASD in Africa can be significantly improved.

## Introduction

Highlights

- Autism in Africa is understudied and there is limited data on the prevalence of the condition. However, studies suggest that the prevalence of autism in Africa is similar to other parts of the world.
- There are significant challenges in diagnosing and treating autism in Africa, including limited access to resources and trained professionals, as well as differences in cultural beliefs and attitudes towards autism.
- Social and cultural factors, such as stigma, discrimination and a lack of awareness, can have a significant impact on families and caregivers of individuals with autism in Africa.

Autism spectrum disorder (ASD) is a multifaceted neurodevelopmental condition that has garnered significant attention and comprehension over the past century^1^. However, there remains a paucity of knowledge about ASD in Africa^2^. The identification and effective management of ASD pose unique challenges to African communities due to limited healthcare resources, poverty and restricted access to mental health services^3^. Moreover, cultural factors significantly influence how ASD is perceived and diagnosed, making it arduous to generalise existing knowledge to the African context^4^. While the body of knowledge about ASD in Africa is growing, much of the available information is outdated, and changes in how Africans view and respond to autism have been modest over the past decade^5^. However, there have been positive developments in recent years. For instance, the establishment of the Centre for Autism Research in Cape Town, South Africa, contributes to research initiatives in Africa and beyond, aiming to raise awareness and comprehension of autism and promote the rights and interests of individuals with ASD and their families^6^. Similarly, the Nigerian government has taken steps to safeguard the rights of individuals with disabilities, including those with ASD^7^. Nonetheless, more work is required to address the unique challenges that African communities face in managing ASD.

Therefore, a comprehensive review of the most recent literature on autism in Africa is essential. Such a review could help identify information gaps and shed light on concerns regarding the prevalence of ASD, its diagnosis, treatment and the impact of social and cultural factors on families and caregivers of individuals with ASD in Africa. Furthermore, the review could inform efforts to develop culturally sensitive diagnostic tools and interventions that can be adapted to the unique needs of African communities.

## Methodology

This review aims to provide an overview of the current knowledge on autism in Africa, specifically focusing on the prevalence, diagnosis, treatment and impact of social and cultural factors on families and caregivers. To achieve this, a comprehensive search was conducted using electronic databases, including PubMed, PsycINFO, Scopus, MEDLINE and Google Scholar. The search strategy employed a combination of relevant keywords and controlled vocabulary terms. The following search terms were used: ‘autism’, ‘autism spectrum disorder’, ‘prevalence’, ‘diagnosis’, ‘treatment’, ‘Africa’, ‘social factors’, ‘cultural factors’, ‘families’, and ‘caregivers’. The search was limited to articles published in English from 2000 to March 2023.

Studies were included if they met the following criteria: focused on autism in African countries, provided information on prevalence, diagnosis, treatment, or the impact of social and cultural factors on families and caregivers, involved human participants and were published in peer-reviewed journals. Grey literature, conference abstracts and studies not available in English were excluded. After removing duplicates, two independent reviewers screened the titles and abstracts of the retrieved articles based on the inclusion and exclusion criteria. The full texts of potentially relevant articles were then assessed for eligibility. Discrepancies in study selection were resolved through discussion and consensus. A total of 55 papers met the inclusion criteria and were included in the final analysis. The selected articles were analysed thematically to identify common themes and patterns related to the prevalence, diagnosis, treatment and impact of social and cultural factors on families and caregivers. The findings were synthesised narratively to provide a comprehensive overview of the current knowledge on autism in Africa.

## Epidemiology of autism in Africaist order

ASD is a global public health concern affecting approximately 1 in 100 children worldwide^8^. However, the prevalence of ASD in Africa is not well-established, with varying estimates reported in different African countries. Lagunju *et al*. reported a prevalence of 2.3% in Nigeria, while Kakooza-Mwesige recorded a prevalence of 0.68% in Uganda^9,10^. Zeglam found a prevalence of 0.33% in Libya, and Hewitt reported a prevalence of 2.07% in Somalia^11,12^. A multinational study that included Egypt and Tunisia reported a prevalence of 33.6 and 11.5%, respectively^13^. However, these varying estimates underscore the need for a more comprehensive and standardised evaluation of ASD prevalence across the continent. The accurate prevalence of ASD is crucial for healthcare providers, policymakers and educators to provide appropriate interventions and services to individuals with ASD and their families^14^. Without an accurate assessment of ASD prevalence, efforts to support individuals with ASD in Africa may be inadequate, and resources may not be properly allocated.

Various studies have investigated the prevalence of ASD in different populations, shedding light on the epidemiology of this condition. Hewitt *et al*.^12^ reported that the prevalence of ASD was higher in White and Somali children in Minneapolis than in Black and Hispanic children. Similarly, Eldin *et al*. conducted a study on Arabic-speaking nations. They found that Egypt had the highest prevalence of ASD (33.6%) among the countries studied, while Tunisia had a prevalence of 11.5%^13,15^. In Nigeria, Oshodi *et al*.^16^ found that 34.5% of the screened individuals had ASD, with learning difficulties, language and speech impairments, intellectual disability and attention deficit hyperactivity disorder being the most commonly associated conditions. Caregivers’ primary concerns included poor language development, gaze avoidance and challenging conduct, while comorbid conditions such as seizure disorders and attention deficit hyperactivity disorder were also observed. The disparities in the prevalence of ASD across different populations and regions may be due to a combination of genetic, environmental and socio-economic factors. These factors may interact in complex ways, making it difficult to establish a clear causal relationship between them and the prevalence of ASD. Nevertheless, research has suggested that genetic factors may play a role in some populations, while environmental and socio-economic factors may be more relevant in others^17^. For example, the higher prevalence of ASD in White and Somali children in Minneapolis than in Black and Hispanic children may reflect genetic differences between these groups and differences in access to healthcare and early intervention services. Similarly, the higher prevalence of ASD in Egypt than in other Arabic-speaking nations may be due to genetic and environmental factors, such as consanguineous marriages and exposure to pesticides. The higher prevalence of ASD in Nigeria may be related to socio-economic factors such as poverty, lack of access to healthcare and education and cultural beliefs. Further research is needed to understand better the complex interplay of genetic, environmental and socio-economic factors that contribute to the disparities in the prevalence of ASD across different populations and regions. This knowledge could help inform the development of more effective prevention and intervention strategies and reduce the burden of ASD on affected individuals and their families.

The estimation of the prevalence of ASD in Africa faces several challenges. One of the most significant obstacles is the limited data on the condition in the continent^18^. This limitation is due to various factors, such as low awareness of the condition, inadequate diagnostic and assessment tools and the stigma associated with mental health disorders^19^. Additionally, the complex nature of ASD, coupled with the limited research capacity in some African countries, poses significant challenges in estimating the prevalence of the condition^20^. Another challenge is the lack of standardised diagnostic criteria and assessment tools for ASD in Africa^21^. Different diagnostic and assessment tools are used across different studies, potentially impacting the reported prevalence rates and making comparing results across different populations difficult. Studies in Egypt and Tunisia used a screening tool based on DSM-IV-TR criteria, while studies in Nigeria and Uganda utilised the Childhood Autism Rating Scale (CARS)^9,10,12^. Such variations in diagnostic criteria and screening tools could potentially influence the prevalence rates of ASD reported in different studies, leading to challenges in comparing prevalence rates across different African countries. Moreover, the absence of culturally appropriate assessment tools for African communities with different languages, values and beliefs further complicates the diagnosis of ASD, contributing to the underdiagnosis of the condition in some African countries^22^.

Furthermore, the high rate of comorbidity in individuals with ASD adds to the complexity of accurately estimating its prevalence. Individuals with ASD often exhibit symptoms of other mental health disorders, making it challenging to differentiate the symptoms of ASD from those of other comorbid conditions^23^. As a result, studies focusing on a particular condition may underestimate the prevalence of ASD. Therefore, to accurately estimate the prevalence of ASD in Africa, there is a pressing need for more comprehensive studies that utilise standardised diagnostic criteria and assessment tools. Furthermore, addressing cultural and social factors that will contribute to the limited research and lower awareness of the condition in some African communities is crucial. These efforts will enable the development of culturally sensitive interventions that could significantly improve the quality of life of individuals with ASD in Africa.

## Diagnosing autism in Africa

The diagnosis of ASD in Africa presents significant challenges due to cultural beliefs, attitudes towards disability and variations in social communication and interaction. To diagnose ASD in Africa, the most commonly used diagnostic tools still include the DSM-5 criteria, the ADOS and the ADI-R^24^. However, Western diagnostic tools are typically based on observing social communication, interaction and repetitive behaviours and interests^25^. However, these criteria often do not accurately capture the full range of behaviours and experiences associated with ASD in African cultures. Cultural beliefs and attitudes towards disability, including mental health disorders, influence the diagnosis of ASD in Africa^3^. Disability is often viewed as a punishment or curse in some cultures, leading families to avoid seeking a diagnosis or treatment for their child^26^. This avoidance is often driven by the fear of social stigma, which may result in isolation and discrimination. Moreover, variations in social communication and interaction in African cultures further complicate the diagnosis of ASD^27^. Direct eye contact and social interactions are not always highly valued in some cultures, while repetitive behaviours and interests may be considered normal^28^. These cultural differences often make it difficult for Western diagnostic tools to capture the full range of behaviours associated with ASD in African cultures.

The resulting underdiagnosis or misdiagnosis of ASD is a common issue in many African countries. It highlights the need for culturally sensitive diagnostic tools that consider the unique cultural beliefs and attitudes towards disability and the variations in social communication and interaction. Such tools would help ensure accurate and timely diagnosis and treatment for individuals with ASD while addressing the social stigma associated with mental health disorders in some African cultures.

The lack of trained professionals is another challenge in diagnosing ASD in Africa. In many African countries, there is a shortage of mental health professionals, and those available are not often trained in diagnosing ASD^29^. This further contributes to the underdiagnosis and misdiagnosis of the condition. Efforts are being made to train more mental health professionals to diagnose ASD, but this remains a significant challenge in many African countries. Various efforts are underway to train more mental health professionals to diagnose ASD. The Kenya Autism Alliance has collaborated with African universities and hospitals to provide training and education in diagnosing and managing ASD^30^. The Kenya Autism Alliance also provides online training programs and workshops for African mental health professionals. Furthermore, developing culturally sensitive diagnostic tools for ASD is another effort to improve the diagnosis of ASD in Africa. These tools aim to incorporate cultural factors and take into account the unique expressions of ASD in different cultures. The South African Autism-Indigenous Screening Instrument (ASAISI) includes culturally relevant items, such as the child’s ability to participate in traditional cultural practices^31^. Similarly, the Nigerian Scale for Autism (NSA) was developed to incorporate Nigerian cultural norms and values^32^. In addition, advocacy and awareness-raising campaigns are being conducted to reduce the stigma associated with mental health disorders, including ASD. These campaigns promote acceptance and understanding of ASD in African communities, encouraging families to seek diagnosis and treatment for affected individuals.

## Treatment of autism in Africa

Behavioural therapies, such as Applied Behavioural Analysis (ABA) and Social Skills Training, are commonly used in Africa to improve the lives of individuals with ASD^33^. ABA is a behavioural therapy that involves breaking down complex tasks into smaller steps and providing positive reinforcement for each step^33^. This therapy improves communication, social and self-help skills in individuals with ASD. Social Skills Training, which utilises role-playing, modelling and feedback to teach and reinforce social skills in a group format, has also demonstrated efficacy in improving social skills and quality of life in individuals with ASD^34^. Cognitive Behavioural Therapy (CBT) is another therapy commonly used in Africa to address negative thought patterns and behaviours in individuals with ASD^35^. CBT typically includes cognitive restructuring, relaxation techniques and exposure therapy. CBT has shown promise in improving the quality of life of individuals with ASD^36^.

Behavioural therapy is integral to the comprehensive treatment plan for individuals with ASD in Africa^37^. This is primarily due to the effectiveness of behavioural therapies in modifying behaviour through positive reinforcement, repetition and consistency, all based on learning theory principles^38^. The heterogeneity of cultural beliefs, attitudes towards disability and variations in social communication and interaction in Africa make diagnosing and treating ASD challenging. Thus, the culturally sensitive approach of behavioural therapies makes them well-suited for the African context.

Behavioural therapies are designed to teach individuals with ASD new skills and behaviours tailored to their specific needs and abilities^39^. These therapies aid in the development of social and communication skills, the management of challenging behaviours and the acquisition of daily living skills. The effectiveness of these therapies has been demonstrated in several studies, highlighting their potential to improve the quality of life of individuals with ASD in Africa^40,41^. Moreover, behavioural therapies are easily adaptable to the cultural context of Africa^42^. They do not rely on Western diagnostic tools and can be delivered by trained professionals or family members who have received proper training. This makes them a cost-effective and accessible option for families and individuals with ASD in Africa^43^. Behavioural therapies have demonstrated the ability to mitigate some of the unique challenges faced by individuals with ASD in Africa, including social stigma and a lack of access to resources^40^.

The use of medication as an adjunct therapy for individuals with ASD in Africa is a topic of growing interest and concern^44^. While psychotropic medications such as antipsychotics, antidepressants and stimulants have been used to address specific symptoms of ASD, the limited research on their efficacy and safety in this population, cultural factors and the shortage of qualified healthcare professionals make using medication challenging^45^. Furthermore, the impact of the cost of medication and poverty in Africa cannot be overlooked when considering medication use in individuals with ASD^46^. The high cost of psychotropic medications, coupled with limited access to financial assistance and mental health resources, contribute to disparities in healthcare and limit access to appropriate treatment for individuals with ASD in Africa^47^.

Given these challenges, it is crucial to approach the use of medication in individuals with ASD in Africa with caution and individualisation. Healthcare professionals must weigh the potential benefits and risks of medication use for each patient while considering the impact of cost and poverty on access to medication and other healthcare services. Addressing the barriers to medication use in individuals with ASD in Africa will require a multifaceted approach that includes improving access to affordable medication, increasing financial assistance for families and improving access to mental health services and resources. By doing so, individuals with ASD in Africa can receive the best care and support for their unique needs.

While evidence-based therapies such as ABA and SST are the first-line approach for individuals with ASD, some families also turn to alternative therapies as complementary or adjunctive treatment^48^. These therapies, including dietary interventions, acupuncture, massage therapy and herbal remedies, are considered nonconventional. While some families report benefits from these treatments, limited scientific evidence supports their efficacy in treating ASD in the African population. Additionally, some alternative therapies carry potential risks and adverse effects, highlighting the importance of discussing their use with a qualified healthcare provider^49^. The use of these therapies also raises concerns about access to evidence-based therapies. Limited resources and healthcare disparities may result in some families turning to alternative therapies as a more accessible option. However, it is crucial to ensure that individuals with ASD have access to evidence-based therapies that are effective in treating their unique needs.

Access to appropriate and effective treatments for individuals with ASD in Africa is a significant challenge, particularly in rural areas where specialised healthcare professionals and resources are limited^50^. Insufficient infrastructure, long waiting lists and delayed diagnosis contribute to inadequate treatment, exacerbating the challenges faced by individuals with ASD and their families^50^. In addition, insufficient awareness and understanding of ASD in the general population create stigma and discrimination, hindering access to essential support and resources^51^. Moreover, the financial burden of treatments and therapies poses a significant challenge to many families, particularly those from low-income communities^46^. This financial burden can limit access to evidence-based interventions, forcing them to rely on alternative therapies with unproven efficacy, which can further exacerbate the challenges of providing appropriate and effective treatment for individuals with ASD.

Culturally appropriate interventions are crucial to addressing the unique needs of individuals with ASD in African settings. The impact of cultural norms, beliefs and practices on health behaviours must be considered to ensure that interventions are acceptable and effective. However, there is a lack of data on the effectiveness of existing interventions, which, coupled with the heterogeneity of ASD presentations across different cultures and populations, complicates the implementation of evidence-based treatments for ASD in African settings. Therefore, there is a pressing need to develop and test culturally appropriate interventions for ASD in African settings, incorporating input from local communities, healthcare providers and individuals with ASD and their families. Such interventions should improve the accessibility, acceptability and effectiveness of treatments for individuals with ASD in Africa, ultimately enhancing their quality of life and promoting greater social inclusion.

## Impact of social and cultural factors on families and caregivers

ASD imposes a considerable emotional and financial burden on families and caregivers in Africa, leading to increased stress levels and limited access to support services^52^ (Table 1). This burden is exacerbated by the disorder’s stigma, leading to social isolation and exclusion^52^. The emotional toll of caring for individuals with ASD can result in high stress, anxiety and depression among family members and caregivers^53^. Furthermore, accessing appropriate treatments and therapies can cause significant financial strain, especially for low-income families^46^. Stigma towards ASD is prevalent in African communities and can make it challenging for individuals with the disorder and their families to access the support services they need^54^. This stigma is perpetuated by cultural beliefs that view mental health and developmental disorders as taboo or caused by supernatural factors. Limited availability and funding of support services, particularly in rural areas, further compound the issue, resulting in delayed or inadequate treatment and exacerbating the burden on families and caregivers.

**Table 1:** Impact of social and cultural factors on families and caregivers.

Social and cultural factors	Impact on families and caregivers
Stigma and social isolation	Increased feelings of social exclusion and discrimination. Limited social support networks. Impact on mental health and well-being.
Lack of community understanding and awareness	Limited understanding of ASD leading to misconceptions and stereotypes. Difficulty accessing support and services. Increased burden on families and caregivers.
Cultural beliefs and practices	Influence treatment-seeking behaviours and acceptance of ASD. Challenges in reconciling traditional beliefs and Western medical approaches. Impact on access to appropriate interventions.
Language and communication barriers	Difficulty in accessing information and services due to language barriers. Challenges in communication and collaboration with professionals. Impact on quality of care and understanding of interventions.

ASD, autism spectrum disorder.

In Africa, cultural beliefs and practices often shape attitudes towards ASD and its treatment^3^. This can lead to delays in seeking medical attention and reliance on alternative treatments. Additionally, cultural beliefs may influence the expectations and goals of treatment, with some families prioritising social integration and conformity over individual expression and development. Various strategies have been employed to promote understanding and awareness of ASD in African communities. One effective approach involves developing and disseminating culturally-tailored educational materials that reflect the unique sociocultural contexts of these communities^54^. Community-based initiatives that engage key stakeholders, including religious leaders, traditional healers and educators, can help to disseminate information and address misconceptions about ASD. In addition, mass media has been used to reach a wider audience and enhance the visibility of ASD^55^. This includes producing television programs, radio shows and print media that feature individuals with ASD and their families, share their experiences and struggles and provide accurate information about the disorder. These campaigns have also sought to counteract the stigma associated with ASD and promote inclusion and acceptance.

ASD advocacy groups and nongovernmental organisations (NGOs) have played a pivotal role in raising awareness and supporting individuals with ASD and their families in Africa^6^. These groups provide a range of services, including counselling, support groups and advocacy and work to build partnerships with governmental and nongovernmental organisations to address the challenges faced by individuals with ASD.

## Recommendations for improving autism care in Africa

To improve autism care in Africa, policy changes are necessary at various levels (Table 2). Governments should prioritise funding for autism care services and establish policies that enable the creation of specialised centres and training healthcare professionals in the field. It is important to provide financial and logistical support for community-based organisations that offer support and advocacy for individuals with autism and their families.

**Table 2:** Challenges and policy recommendations of ASD care in Africa.

Challenges	Policy recommendations
Limited awareness and education	Develop public awareness campaigns about ASD, its early signs, and available services. Include ASD education in school curricula and provide training for teachers and healthcare professionals. Establish community-based programs to disseminate information about ASD to families and communities.
Inadequate access to diagnosis and assessment services	Strengthen and expand ASD diagnostic services, including specialized clinics and trained professionals. Reduce diagnostic waiting times and improve access to early intervention services. Establish referral networks and partnerships between healthcare providers and community organisations.
Limited availability of evidence-based interventions	Promote the training and certification of professionals in evidence-based interventions such as Applied Behaviour Analysis (ABA) and cognitive behavioural therapy (CBT). Support the adaptation and translation of evidence-based interventions to local cultural contexts. Invest in research to evaluate the effectiveness of interventions in African settings.
Financial barriers and limited insurance coverage	Advocate for the inclusion of ASD-related services in national health insurance schemes. Develop subsidies or financial assistance programs to support families in accessing ASD care. Strengthen collaborations between government, non-profit organisations, and private sector stakeholders to fund ASD services.
Stigma and social isolation	Implement anti-stigma campaigns to reduce social stigma and discrimination against individuals with ASD and their families. Promote inclusive education and community integration for individuals with ASD. Establish support groups and community-based initiatives to foster social support and inclusion.
Limited research and data collection	Encourage research collaborations and partnerships between African countries and international research institutions. Invest in national and regional ASD registries or databases to collect data on prevalence, intervention outcomes, and service utilisation. Allocate funding for research on ASD causes, risk factors, and effective interventions in the African context.

ASD, autism spectrum disorder.

Stakeholder involvement is critical in developing and implementing policies related to autism care. Collaboration between healthcare providers, individuals with autism and their families, advocacy groups and other relevant organisations can help to ensure that policies and interventions are culturally appropriate, effective and sustainable. Integrating autism care into existing health systems is also essential and can be achieved through referral networks that allow for the seamless transfer of patients between different levels of care. Additionally, governments should prioritise including autism care in national health insurance schemes to increase access to affordable services.

Efforts to increase public awareness and reduce the stigma around autism in African communities are also necessary. Education and awareness-raising initiatives should target healthcare providers, educators and the general public to promote early detection, diagnosis and treatment of autism. Media campaigns, public events and community outreach programs can effectively achieve this goal.

## Conclusion

The current state of knowledge on autism in Africa highlights the pressing need for more research, resources and policy changes to address the unique challenges faced by individuals with autism and their families in this context. Despite the significant gaps in data and resources, progress has been made in recent years, with increased attention to autism at the national and international levels and efforts to improve awareness and access to services. However, much work remains to ensure that individuals with autism in Africa have access to appropriate and effective diagnosis, treatment and support.

The high prevalence of autism in Africa and the limited resources available to address this issue underscore the urgency of this matter. The challenges families and caregivers face, such as stigma, lack of awareness, and limited access to services, are significant and require a multifaceted approach considering the social, cultural, and economic context in which they occur. This includes efforts to improve public awareness and education, expand access to evidence-based interventions and invest in research and policy changes that can improve the lives of individuals with autism and their families.

Building partnerships between healthcare providers, researchers, policymakers and communities is critical to develop and implement sustainable solutions that meet the unique needs of individuals with autism in Africa. By working together, Africa can progress in addressing the challenges faced by individuals with autism and their families in Africa and ensure they have access to the care and support they need to thrive.

## Ethical approval

Not applicable.

## Consent

Not applicable.

## Sources of funding

No external funding was received for this study.

## Author contribution

N.A.: conceptualization. All authors participated in the writing of the paper.

## Conflicts of interest disclosure

The authors declare no conflicts of interest.

## Research registration unique identifying number (UIN)

1. Name of the registry: not applicable.
2. Unique identifying number or registration ID: not applicable.
3. Hyperlink to your specific registration (must be publicly accessible and will be checked): not applicable.

## Guarantor

Nicholas Aderinto.

## Provenance and peer review

Not commissioned, externally peer-reviewed.

## Data availability statement

Not applicable.
